# Comparative study of flocculation and adsorption behaviour of water treatment proteins from *Moringa peregrina* and *Moringa oleifera* seeds

**DOI:** 10.1038/s41598-019-54069-2

**Published:** 2019-11-29

**Authors:** Shirin Nouhi, Habauka M. Kwaambwa, Philipp Gutfreund, Adrian R. Rennie

**Affiliations:** 1 Centre for Neutron Scattering, Uppsala University, Box 516, 751 20 Uppsala, Sweden; 2 Present Address: Swerim AB, Box 7047, 16407 Kista, Sweden; 3 Namibia University of Science and Technology, Faculty of Health and Applied Sciences, Private Bag 13388, 13 Jackson Kaujeua Street, Windhoek, Namibia; 4 Institut Laue - Langevin, 71 avenue des Martyrs, F-38000 Grenoble, France

**Keywords:** Physical chemistry, Colloids, Chemical engineering, Environmental sciences

## Abstract

Trees of *Moringa oleifera* are the most widely exploited species of *Moringa* and proteins extracted from its seeds have been identified as the most efficient natural coagulant for water purification. Largely for climatic reasons, other *Moringa* species are more accessible in some regions and this paper presents a comparative study of the adsorption to different materials of the proteins extracted from seeds of *Moringa peregrina* and *Moringa oleifera* to explore their use as flocculating agents in regions where each is more readily accessible. Results showed that *Moringa peregrina* seed proteins had higher adsorption to alumina compared to silica, in contrast to opposite behavior for *Moringa oleifera*. Both species provide cationic proteins that can act as effective coagulants for the various impurities with different surface potential. Despite the considerable similarity of the amino acid composition, the seed proteins have significantly different adsorption and this presents the opportunity to improve processes by choosing the optimal species or combination of species depending on the type of impurity or possible development of separation processes.

## Introduction

## Challenges in Water Purification

Remediation and purification of water remain a major challenge for environmental engineering in many areas: there is a continuing pressure to provide safer water supplies. Costs must be carefully controlled, and it is highly desirable to avoid the use of treatment chemicals that could be toxic if used incorrectly or in excess. Furthermore, there is a need to develop processes that do not require the supervision of trained technical personnel particularly in rural areas in developing countries. These reasons are some of the powerful drivers to investigate the exploitation of non-traditional technology in water purification.

In treatment of water for human consumption or the remediation of wastewater, a common first step is clarification by coagulation or flocculation of particulate impurities that can then settle or float according to their density. This process is important both to remove potentially toxic material but also to enable further stages such as disinfection where the required amounts of oxidizing agents such as chlorine or ozone would be substantially increased if there were too much organic residue. Even ultraviolet irradiation requires adequate water clarity. Many specifications for water treatment, therefore, impose standards of sufficiently low turbidity not only because discolored water is unattractive.

The process of flocculation of the impurities has been described extensively^1^ and involves controlled stirring and reduction the stability of particles. Often particulate impurities are negatively charged. In brief, colloidal stability is usually reduced by adding polyvalent salts such as aluminum sulfate or iron (III) chloride^2^, or by adding synthetic polymers that are often cationic^3^. The mechanism in the first case depends on screening the charged repulsion that imparts colloidal stability to the particles. In the latter case, the polymers are chosen to adsorb readily and are usually sufficiently large that they may link to several particles. Choice of flocculating agents and mixing conditions that produce large flocs that are compact or can be dewatered readily is valuable for efficient processing.

Difficulties with conventional treatment arise from potential toxicity if high concentrations of metal ions remain in the water. For example, aluminum has been implicated in Alzheimer’s disease^4–6^. In the case of iron salts, it is often necessary to precipitate excess by increasing the pH. Cationic polymers are regarded as environmental pollutants that are toxic to fish^7^ and so residues in treated water need to be minimized. For these reasons, there has been increasing attention to find alternative flocculants particularly from natural products. A range of biopolymers have been identified that include polysaccharides and proteins^8–12^ as potentially useful, however, feasibility of use of particular products is affected by lack of availability in some areas.

## Background

The most widely exploited natural material for water treatment has been crushed seeds from *Moringa oleifera* trees^13^. This use was first observed as a traditional process in the valley of the Nile river but work by Broin *et al*.^14^, for instance, identified that the protein from the seeds is the effective coagulant. Adsorption to various materials has been reported^14–18^ and it was shown that although the molecules are small, they bind effectively to many potential impurities as well as self-associate so that flocculation occurs. The material effectively clarifies water with impurities that include mineral particles, bacteria and algae^19–21^. Engineering tests have been made with various scales of process equipment from small scale jar-tests^22^ up to plant trials (e.g.^23,24^).

Although other seed proteins could be exploited to some extent in a similar manner, systematic comparison of over a hundred natural coagulants has shown that those of *Moringa oleifera* trees are the most effective^8,9,25–28^. These seeds are also a rich source of oil and the widespread use of different parts of the tree for nutrition and medicine in many countries gives ready acceptance for use in the treatment of drinking water^29^. *Moringa* seed proteins have therefore been proposed for wider use as a biodegradable, low-toxicity, low-cost and sustainable replacements to the usual synthetic materials and they can be used in a simple way without trained technical supervision^30^.

There are thirteen known species of *Moringa* trees that are native to Africa, Asia, Middle East and Madagascar^31–33^ but some are now distributed widely in various regions of the world, including areas with little rainfall. Although *Moringa oleifera* is the most widely used, it is interesting to consider the possibility to exploit other varieties. There is much less known about the alternative species and there are few comparative studies showing whether they can be as effective as *Moringa oleifera* in water treatment. The species native to East Africa, *Moringa stenopetala*, has been investigated^34,35^ however several papers related to water treatment have concerned uses other than as a coagulant such as to bind heavy metals^36,37^. In respect of the *Moringa oleifera*, there have been several reports that the proteins are a complex mixture. Moulin *et al*.^38^ have reported extensive mass-spectrometry studies of the material that was obtained from Africa and the study clarified that certain components may bind differently to various materials.

*Moringa peregrina* is another widely grown species and it is native to various regions such as Iran, the Arabian Peninsula, India, parts of Africa and as far north as the Dead Sea^39^. This geographical spread includes many areas where populations suffer from limited access to clean water supplies. In the present study, we have chosen seeds from two different varieties of *Moringa* trees from different regions, to explore their use as a flocculating agent, and to correlate that with molecular properties and the adsorption of the proteins to different materials.

## Design of the Experiment

The adsorption of proteins has been studied at three types of surfaces with different surface charge and properties, so that the efficiency of seed proteins as flocculating agents for various impurities could be investigated. Although purified proteins were used to identify differences between samples, the extraction procedure, e.g. using cold water without added salt, was chosen so that the samples would resemble those that would be used in practical applications. It is known that the extracted material obtained with high concentrations of salt or with hot water can be different^40–42^. Surfaces chosen for this study were polystyrene colloidal particles and flat silica and alumina surfaces. These provide model systems of interfaces with different properties but characteristics which can be relevant for water purification. Silica and alumina are models for mineral particles that can be found as impurities in water. At neutral pH, silica carries a negative charge when immersed in water whereas alumina is neutral or slightly positive^43^. Their comparison allows exploration of the effect of surface charge on the adsorption of proteins. Polystyrene latex used in this study provides an example of a heterogeneous interface with both hydrophobic regions and some groups that carry negative charge. It provides a model for typical particles dispersed in water. Neutron reflectometry was chosen as a technique that allows determination of the structure and composition of interfacial layers as well as the amount of material bound to the surface^44^. The reflected intensity measured as a function of wavelength and angle at low grazing angles can be analyzed to provide the molecular composition profile. Neutron reflection is valuable for studies of adsorption as it is straightforward to apply, and the results are simple to interpret. However, it requires the use of large, flat substrates that are transparent to neutrons so that the signal reflected at low grazing angles of incidence can be measured. The technique is sensitive to the adsorbed layer onto the surface (see Figs. S1 and S2 in Supporting Information). Both silicon crystals, which forms a native silica oxide layer, and alumina (sapphire) have low absorption of neutrons and are readily available as large and flat substrates. This provides another motivation to use silica and alumina for the adsorption behavior of proteins in this study. The flocculation caused by the seed proteins was tested with a dispersion of polystyrene latex particles; and the transmission of visible light through the sample was measured. The zeta potentials of the proteins were measured, and the amino acid compositions of the different samples were determined.

## Experimental

### Materials

**Figure 1:**
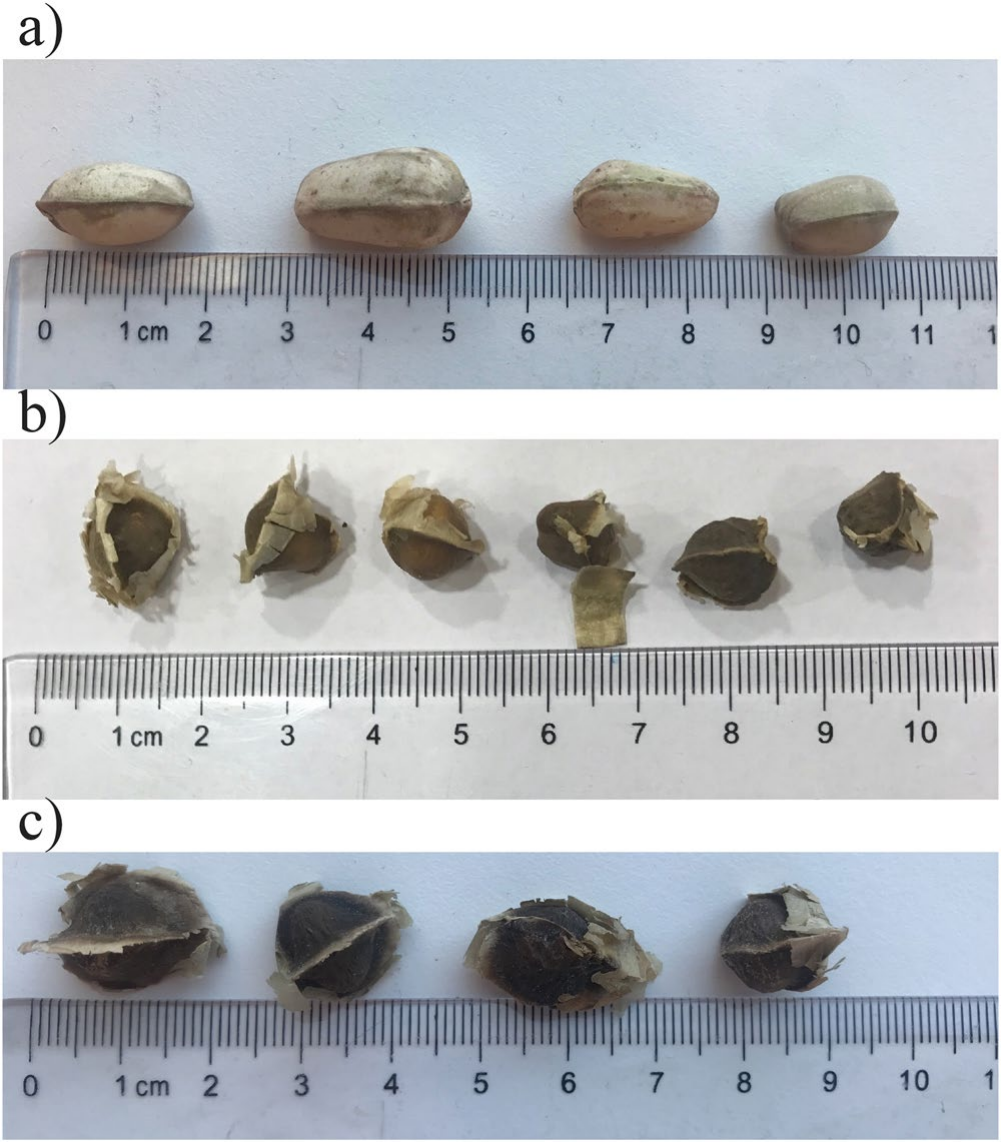
*Moringa* seeds used in this study include *Moringa peregrina* (**a**), *Moringa* oleifera from Iran (**b**) and *Moringa* oleifera from Botswana (**c**).

The seeds used for this study were provided by local suppliers in Iran and Africa. Seeds of *Moringa oleifera* from Africa were collected from Botswana, Iranian *Moringa oleifera* came from Bushehr, Bushehr province, and the *Moringa peregrina* from Nikshahr, province of Sistan and Baluchestan. *Moringa* seeds are obtained from the pods of the tree, each pod normally contains 10–12 seeds. Seeds are large and contain between 20–50% proteins, providing an abundant source of proteins^35^. Figure 1 shows a picture of the seeds before deshelling. Seeds of *Moringa oleifera* from Iran and Botswana are both winged and look very similar, whereas *Moringa peregrina* seeds have an unwinged hard shell. The *Moringa oleifera* seed protein from Botswana was the same sample as that used in previous studies^18^.

The seeds were stored at room temperature prior to the extraction of the proteins. The extraction procedure was similar for all samples and started by deshelling and crushing the seeds using a kitchen blender. For the *Moringa peregrina* because of the consistency of the oily product, it was necessary to use a pestle and mortar as well. Crushed seeds were then mixed with petroleum ether, 40–60 °C, (1:5 ratio) and stirred on a magnetic stirrer for about 2 hours to dissolve the oil from the seeds. The paste was separated using vacuum filtration and the resulting powder was dried in air overnight. The protein was then dissolved in deionized water (each 50 g in about 200 mL water) using a magnetic stirrer for about an hour and the solution was filtered and the solid washed using 2–3 times more pure water until a relatively clear solution was obtained. The proteins were then precipitated from the solution as a sticky paste by adding ammonium sulfate to saturation. The paste was redissolved in deionized water and filtered again. The solution was dialyzed against high purity water using cellulose membrane tubing (Sigma-Aldrich D9527-100FT, molecular weight cut-off 14 kDa) to remove ammonium sulfate. To further purify the proteins, dialyzed solution was added to a carboxymethyl cellulose packed column, to which the proteins bind. The bound proteins were then released and eluted with a 1 M NaCl solution. Eluted solution was dialyzed using dialysis tubing as above in order to remove the remaining salt and the protein powder was finally obtained by freeze drying.

A further small difference in the extraction procedure was the type of cellulose column used to purify the protein. Proteins from the seeds of *Moringa oleifera* from Botswana were extracted previously using CM-Cellulose microgranular 25–60 μm purchased from Sigma-Aldrich (C4021) which is no longer available, whereas the proteins from seeds from Iranian, used Ionsep CMC 52 pre-swollen carboxymethyl cellulose was purchased from Biophoretics.

Charge stabilized polystyrene colloidal particles with 720 Å radius (polydispersity < 1%), PS3, were used as a model system for an impurity in water. The particles were synthesized and characterized as described in previous articles^19,45^. The surface potential of the particles was determined to be about −30 mV at pH 7.

Substrates for this experiment were a 5 cm × 5 cm × 1 cm crystal of sapphire (alumina, Al_2_O_3_) and the amorphous silica (SiO_2_) layer formed on a silicon crystal. The crystals were cleaned prior to the experiment by immersing in diluted Decon90 in a clean Petri dish for a few minutes and then rinsing with Milli-Q water. The cleaning was continued by spreading drops of concentrated sulfuric acid over the reflecting surface of the crystal and adding approximately the same amount of water. After leaving for a few minutes, the surface was then rinsed extensively with water. Cleaning with acid was repeated two or three times until the surface of the crystal was clearly hydrophilic with no measurable contact angle for water.

### Methods and procedures

The zeta potential was measured for different proteins concentration in water using a Malvern Zetasizer Nano ZS in disposable cells using the dip accessory, ZEN1002. Analysis of amino acid composition was made by Alphalyse, Denmark. Details of the procedure are provided in the supporting information.

Flocculation of particles caused by the proteins was tested by measuring the ultraviolet (UV) transmission spectra of dispersions of polystyrene latex particles in water both in the absence and presence of *Moringa* seed proteins, using a Lambda 35 UV/VIS spectrophotometer. The sample holders for the UV transmission measurements were 1 mm path length fused quartz cuvettes as shown later in results. Spectroscopy data is commonly shown as transmission of the beam through the sample at different wavelengths. According to the Beer-Lambert law, transmission (${\rm{\tau }}$) is defined as:

1 $$\tau =\frac{{\rm{I}}}{{{\rm{I}}}_{{\rm{o}}}}=\frac{{\rm{I}}}{\in \,{\rm{.l}}{\rm{.c}}}$$

where *ϵ* is the absorptivity, *l* is the path length of the beam and *c* is the concentration of the sample, *I* is the intensity of the transmitted beam and *I*_*o*_ is the incoming beam.

The amount and the structure of the proteins adsorbed to different surfaces were determined as a function of the solution concentration using neutron reflectometry. In this experiment, the intensity of the reflected beam was measured at a specific angle (where the angle of incoming and outgoing beam is the same) as a function of wavelength with the D17 instrument at the Institut Laue Langevin, Grenoble, France^46^. The reflected intensities for different interfacial structural models were calculated and fitted to the data using a least squares minimization to obtain the best fits^47^.

Sample holders used for the neutron reflectometry experiment are described in detail by Rennie *et al*.^48^. In this design, the sample was contained by a 2 mm thick polytetrafluoroethylene (PTFE) gasket between two crystals. This allows measurements of the same solution with two different surfaces by rotation and translation to bring the different interfaces into alignment. The crystals were clamped together with an aluminum frame that has channels for circulating water that allows control of the sample temperature. For these measurements, samples were kept at 25 °C. Data are recorded as the ratio of the reflected intensity to that of the incident beam as a function of Q that is given by Q = (4π/λ) sin θ.

The stock protein solutions were prepared at 0.1 or 0.2 wt. % concentration in water (highest measured concentration) by tumbling overnight. The pH (pD, corresponding to the samples prepared in D_2_O for neutron reflection experiment) of 0.2 wt% solutions were found to be in the range 6 to 6.8. The stock solutions were diluted to the desired concentrations during the neutron experiment using a Knauer HPLC pump with a flow rate of 2 mL min^−1^.

## Results and Discussion

### Flocculation of particles

**Figure 2:**
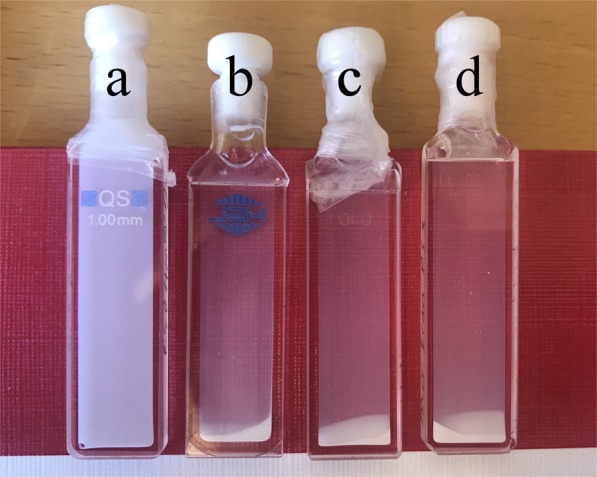
0.5 wt. % PS3 latex particles in water in the absence of *Moringa* seed proteins (**a**) and mixed with 0.2 wt. % of *Moringa peregrina* seed proteins (**b**), *Moringa* oleifera seed proteins from Iran (**c**), and *Moringa* oleifera seed proteins from Botswana (**d**).

The dispersion of 0.5 wt. % PS3 latex particles in deionized water with no additive is shown in Fig. 2 (labelled as **a**) together with the other samples that also contain 0.5 wt. % of particles dispersed in aqueous solutions with added 0.2 wt. % of the different *Moringa* seed proteins. The picture is taken at about 10–15 minutes after mixing. Sedimentation of particles due to flocculation by *Moringa* seed proteins is evident for the samples **b** to **d** and appears by eye similar for the different *Moringa* seed proteins used in this study.

**Figure 3:**
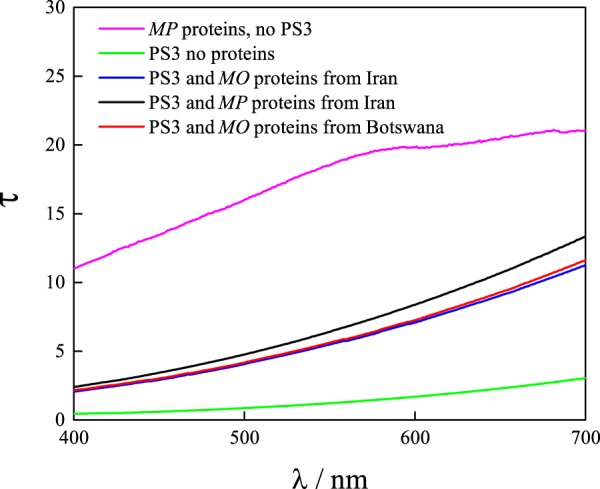
Visible light transmission measured for the dispersion of 0.5 wt. % of PS3 particles, 0.2 wt. % *Moringa peregrina* (MP), and a mixture of different *Moringa* seed proteins species with particles in water. MO stands for *Moringa* oleifera.

To make a quantitative comparison of the flocculation of particles, transmission spectra for visible light were recorded for the samples shown in Fig. 2 and these are shown in Fig. 3. The transmittance of dispersions with different *Moringa* seed proteins increased by more than a factor of five from the initial values for all three types of seed proteins at a wavelength of 650 nm. This suggests that the efficiency of *Moringa peregrina* seed protein as a flocculating agent is as high as *Moringa oleifera* seed proteins. The results also showed that for the *Moringa oleifera* grown in different regions, flocculation of latex is similar.

### Amount and structure of bound protein layers

**Figure 4:**
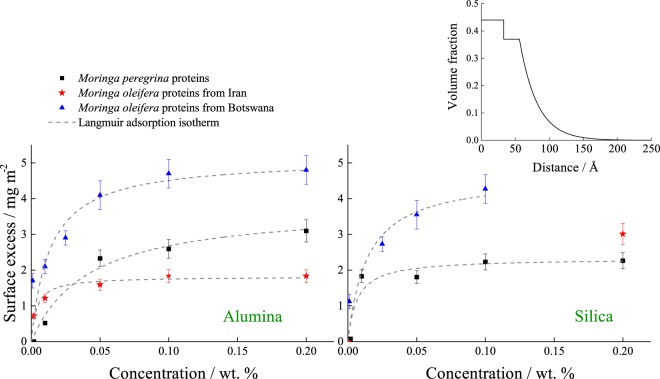
The bound amount (surface excess) for protein from *Moringa peregrina*, and *Moringa* oleifera seeds from Iran and Botswana on alumina (left) and silica (right) surfaces determined from the neutron reflection data. The adsorbed amount of *Moringa* oleifera seed proteins from Botswana on silica is taken from the work of Nouhi *et al*.^53^ and on alumina from Kwaambwa *et al*.^17^. The inset shows the density profile for *Moringa peregrina* on silica at 0.2 wt%.

The amount of protein bound to the two surfaces at various protein concentrations derived from fits to the neutron reflection data is shown in Fig. 4. All proteins adsorbed to both alumina and silica surfaces and the bound amount reaches a plateau at concentrations of about 0.1 wt. %. Seed proteins from *Moringa oleifera* grown in Botswana showed higher adsorption on both surfaces. At the same concentrations, *Moringa peregrina* proteins showed more adsorption to alumina than to silica. The *Moringa oleifera* seed proteins from the trees grown in Iran showed higher adsorption to silica at the single concentration that could be measured in the allocated beam time, than to the alumina but overall amounts bound to both surfaces were lower than for the sample from Botswana. This provides ideas as to which species could be more effective for aggregating certain types of impurity in water.

The adsorption was compared by fitting the data to a Langmuir adsorption isotherm, which is commonly used as a simple model. The results are shown in Table S2, Supporting Information. At the alumina surface, *Moringa oleifera* from Iran shows a significantly higher Langmuir adsorption constant but lower maximum absorbed amount compared to that of *Moringa oleifera* from Botswana.

The best fitting models to the neutron reflectivity data (Fig. S3, Supporting Information) showed that the adsorption of proteins to both silica and alumina surfaces could be described with a dense protein layer close to the solid surface and a further region with an exponential decay of concentration towards that in the bulk solution. The inset plot on the top right of Fig. 4 shows an example of the density profile of the protein layers adsorbed to the solid surface as a function of distance from the interface. It has been shown previously extracts from the seeds of *Moringa oleifera* contain several distinct proteins^17,49^, which is likely to be the origin of the observed high adsorption in the form of a multilayer to solid surfaces^49^. Multilayer adsorption was also found in this study for the proteins extracted from the seeds of *Moringa peregrina*, suggesting that the extract proteins contain several proteins. It has been shown that the proteins extracted from seeds can vary according to the specific extraction procedure, explicit descriptions of various materials with different molecular mass have been described previously^50–53^. There are small differences, for example in the amount of material bound to silica^17,53^ that have been reported for samples of *Moringa oleifera* extracted with the same procedure. However, the overall pattern of adsorption and coagulation behavior are normally the same.

These results revealed that proteins from seeds of *Moringa peregrina* form a denser layer at the alumina surface (70% vol. proteins) compared to the silica surface (50% vol.), whereas the *Moringa oleifera* seed proteins from Iran tree have a denser layer on the silica surface (70% vol.) surface than the alumina surface (50% vol.). For the seed proteins from both *Moringa oleifera* and *Moringa peregrina* collected from Iran, the width of the decaying density profile was twice as large on alumina (~20 Å) as compared to silica (~10 Å). The different structure observed for these materials might arise from different compositions in the samples with some specific protein molecules binding more to different surfaces.

The structure of the layer close to the surface for *Moringa oleifera* seed proteins from Iran and Botswana were very similar, however, the seed proteins from the latter were observed to have a slightly thicker dense layer on both surfaces and a region with a decaying density profile that was nearly twice as wide on the silica surface. This suggests that these differences in source material might alter the capability to form multilayers and thus change the amount required to give rise to saturated coverage of a particular surface.

For the effect of rinsing with water for the three samples of *Moringa* seed proteins, the reflectivity was studied by rinsing the surface that had been exposed to the highest concentration of *Moringa* seed proteins with water. The cell was flushed with about 30 mL of water (>10 times volume of the cell). The reflectivity did not change from that seen in the presence of the solution from the adsorbed layer at the highest concentration after rinsing (see Fig. S5 in Supporting Information). This indicates that the protein layer is irreversibly bound to the interface or would require some other chemical change in the solution to displace the adsorbed material. It has been shown previously that an adsorbed layer of *Moringa oleifera* proteins can be removed from the surface by rinsing with a solution of cationic surfactant, hexadecyltrimethylammonium bromide (C_16_TAB)^18^. Further studies are required to understand and compare the removal of *Moringa oleifera* and *peregrina* seed proteins from solid surfaces.

### Molecular and surface potentials

As mentioned above, the interfacial potentials for alumina and silica in pure water are known to be different^42^. To understand the role of charge effects on binding, zeta potentials for all the proteins were measured at different concentrations in H_2_O and they were all found to be +20 ± 5 mV (see Fig. S4 in Supporting Information). The positive zeta potential suggests that the differences observed in the adsorption behavior is unlikely to be simply due to the overall surface charge but other binding mechanisms and chemical influences could be important.

### Protein amino acid composition

**Figure 5:**
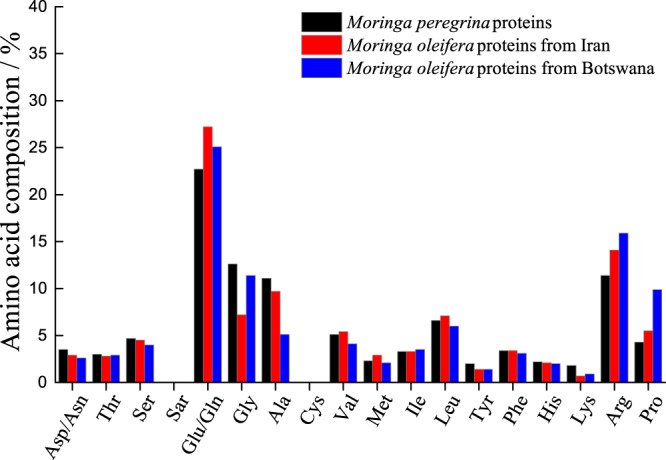
The amino acid composition for samples of the different *Moringa* seed proteins used in this study.

Several studies have reported either the amino acid composition of *Moringa oleifera* seed proteins^17,49,50,54,55^ or the sequence of some specific molecules. There has also been one report of the composition for *Moringa peregrina*^30^. There are some differences in the reported compositions that may be due to region, harvesting conditions, climate, and/or the extraction and purification process. The amino acid composition determined for the different species of *Moringa* seed proteins used in this study is shown in Fig. 5.

Noticeable differences in the amino acid composition are seen for glycine, glutamic acid/glutamine, alanine, arginine and proline (with a maximum of 5% difference). Arginine is positively charged and glutamic acid is negatively charged at neutral pH. Seed proteins from *Moringa oleifera* grown in Iran showed slightly lower arginine and higher glutamic acid/glutamine compared to that grown in Africa (2–3%). This may explain the higher adsorption of the proteins from trees grown in Botswana^18^ compared to that of the trees grown in Iran. Seed proteins of *Moringa oleifera* and *Moringa peregrina* from the trees grown in Iran, show a difference in the fraction of arginine residues. *Moringa oleifera* seed proteins show a higher fraction of arginine which can explain higher adsorption to silica compared to *Moringa peregrina*. There are reports of different amounts of overall oil and protein as well as differences in composition for *Moringa peregrina* in a study evaluating its use as a food source^56^.

There is a clear possibility that these small changes in amino acid composition can be responsible for the differences seen in the adsorbed amount and the structure of seed proteins adsorbed to different surfaces. However, there is also a possibility that there are other differences, for example, in the sequence of the proteins that would not be identified by this simple analysis. It has been shown that different sequences of individual *Moringa oleifera* seed proteins with differences in only a few amino acid residues that were separated from a crude extract can cause significantly different adsorption properties^49^. To test whether these effects arise from differences between species, growth conditions or other factors and to understand these at the molecular level would require a much larger variety of seed samples grown under controlled conditions as well as further work to fractionate the components.

## Summary

The present study provides a comparison between the interactions of proteins extracted from seeds of *Moringa peregrina* and *Moringa oleifera* tree, with model mineral surfaces and colloidal particles. *Moringa oleifera* seed proteins have been suggested as the most effective natural coagulant/flocculant for water purification purposes, however, they only grow in specific regions. *Moringa peregrina* grows as native species in different regions of the world but has been less exploited as a coagulant. The results of this study show that *Moringa peregrina* seed proteins can provide an effective flocculent for colloidal particles and that it acts in a similar way to *Moringa oleifera* seed proteins in the clarification of water.

Understanding the adsorption and association can help finding an effective aggregation agent native to some regions without having to access a specific species or produce what is needed for a specific purpose and develop the purification technologies by modifying the species of the plants or their growing conditions.

*Moringa peregrina* seed proteins have broadly similar surface charge and amino acid compositions to *Moringa oleifera* seed proteins. The extracted material consists of several proteins and adsorbs to mineral surfaces as a multilayer, in a similar way to *Moringa oleifera* seed proteins. Despite the similarities, there are interesting differences such as in the adsorbed amount and the structure of layers at solid surfaces. Seed proteins of *Moringa peregrina* offer unique adsorption properties as they bind more to an alumina surface than a silica surface. This could pave the way towards developing new methods for purifying certain impurities which cannot be removed as effectively using *Moringa oleifera* seed proteins and separation procedures might be developed or use of the optimum mixture of different proteins could increase the efficiency of aggregation. Just like for *Moringa oleifera* seed proteins, the adsorption of *Moringa peregrina* seed proteins onto mineral surfaces reaches a plateau at a concentration of approximately 0.1 wt. %. This knowledge of the amount needed in solution helps to optimize the amount used and avoid unnecessary residual material being left in processed water.

Seed proteins from same species grown in different regions also showed a different adsorption behavior, i.e. thicker layer with a longer decaying length was observed for the protein of *Moringa* oleifera seeds grown in Botswana. This suggests that by modifying the trees and/or the growing conditions, proteins with the most effective properties can be obtained. However, to achieve this goal, further studies are required to understand and distinguish between the various different effects, for example how the properties of seeds can vary between different trees grown on the same field, the same tree grown in different conditions, harvesting season, extraction procedure, etc.

## Supplementary information

Supporting information
