# Supplementary material for: Comparative study of flocculation and adsorption behaviour of water treatment proteins from Moringa peregrina and Moringa oleifera seeds
Source: Sci Rep. 2019 Nov 29;9:17945. doi: 10.1038/s41598-019-54069-2 (PMC6884452; doi:10.1038/s41598-019-54069-2)
Supplement: Supplementary file 1 — Supporting information [file 41598_2019_54069_MOESM1_ESM.pdf]

**Supporting information**

**Comparative study of flocculation and adsorption behaviour of water treatment proteins from *Moringa peregrina* and *Moringa oleifera* seeds**

Shirin Nouhi[1,2*], Habauka M. Kwaambwa[3], Philipp Gutfreund[4] and Adrian R. Rennie[1]

1. Centre for Neutron Scattering, Uppsala University, Box 516, 751 20, Uppsala, Sweden.
2. Current address: Swerim AB, Box 7047, 16407 Kista, Sweden.
3. Namibia University of Science and Technology, Faculty of Health and Applied Sciences, Private Bag 13388, 13 Jackson Kaunjeua Street, Windhoek, Namibia.
4. Institut Laue - Langevin, 71 avenue des Martyrs, F-38000 Grenoble, France

* Corresponding author, E-mail address: shirin.nouhi@swerim.se

**Details of amino acid analysis**

The acid hydrolysis is performed at 110°C in 6 M HCl, 0.1% phenol and 0.1% thioglycolic acid under reduced pressure in an argon atmosphere. The amino acid separation was performed using ion exchange chromatography, the derivatization with ninhydrin, and the detection of various components at 570 and 440 nm. As an internal control, the sample was prepared with a known amount of sarcosine. The method used did not identify tryptophan and cysteine. Asparagine is included as aspartic acid, and glutamine as glutamic acid. Degradation of methionine, serine and threonine could reduce the determined fractions by 10% of the reported values but significant differences were not reported for these components.

**Neutron reflectometry experiment**

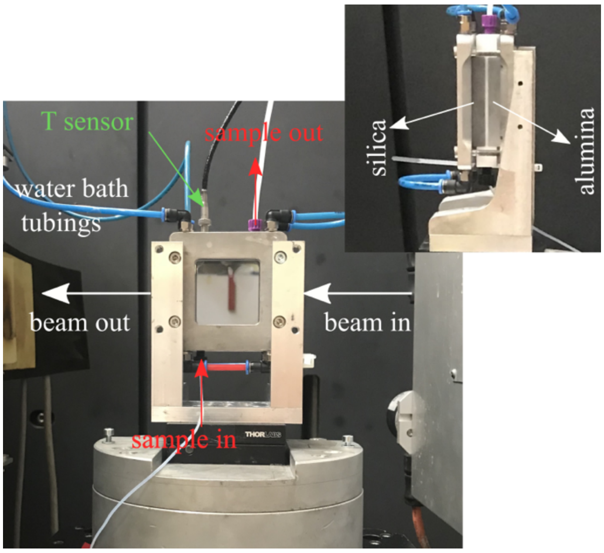


Figure S1a. Neutron reflectometry set up on D17, ILL, Grenoble: top right figure shows a side view of the cell showing the two crystals separated by the PTFE gasket.

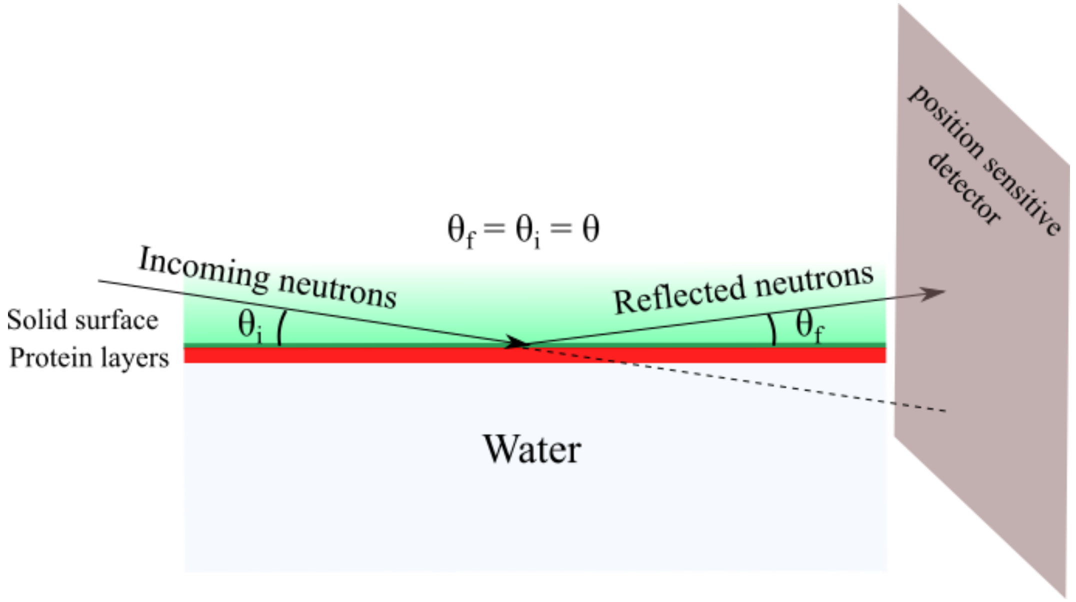


Figure S1b. A schematic representation of neutron reflectometry experiment, where $\theta_i = \theta_f\ (=\theta)$. The reflection is determined as the ratio of the intensities of the reflected and incident beam.

The characteristic amount and the structure of *Moringa* seed proteins adsorbed to different surfaces in this article were studied using neutron reflectometry where the measured signal is highly sensitive to the adsorbed amount at the interface [1]. Neutrons can penetrate deep into some materials such as silicon or sapphire and this can make them a very powerful tool to study the structures at solid/liquid or so-called buried interfaces. The neutron reflectometry measurements for this study were carried out D17 reflectometer, at Institut Laue-Langevin (ILL), Grenoble, France. Figure S1 shows a photograph of the neutron reflectometry setup on D17 with various components of sample holder marked on the image, and the top right image is a side view of the sample holder showing the sample sealed between the two silica and alumina surfaces. Figure S2 shows a schematic representation of a neutron reflectivity measurement experiment, where neutrons are illuminated to the interface from the solid surface that is nearly transparent to neutrons. The top figure shows the signal measured from a bare silicon substrate with a native oxide layer exposed to water and the bottom figure shows the signal measured when layers of in this case proteins are adsorbed to the surface. Neutrons are sensitive to isotopes, hence show very different scattering potential for $H_2O$ and $D_2O$. This can be very useful to study the structure of adsorbed proteins since $H_2O$, $D_2O$ or a mixture with correct ratio can be used interchangeably to enhance the contrast and highlight the signal from the adsorbed layer.

**Interpretation of neutron reflectometry data**

Neutron reflectivity is a measure of the ratio between the intensity of the reflected beam and the incoming beam in the specular condition ($\theta_i = \theta_f$), where $\theta_i$ is the angle of the incoming beam

and $\theta_f$ is the angle of the outgoing beam. It can provide information on the thickness and composition of the layers adsorbed at the interface. Reflectivity is calculated using optical methods with a recursive matrix algorithm dividing the structure into layers of defined refractive index and thickness. The refractive index of a material for neutrons is defined by a term called scattering length density. Scattering length density is calculated as:

$$\rho = \Sigma b_i / V \quad (1)$$

where $b_i$, the scattering length, is a characteristic of each atom which depends on the strength of its interactions with neutrons, and the sum is taken over all of the elements found in the volume V. See Table S1 for scattering length densities of the material used in this experiment.

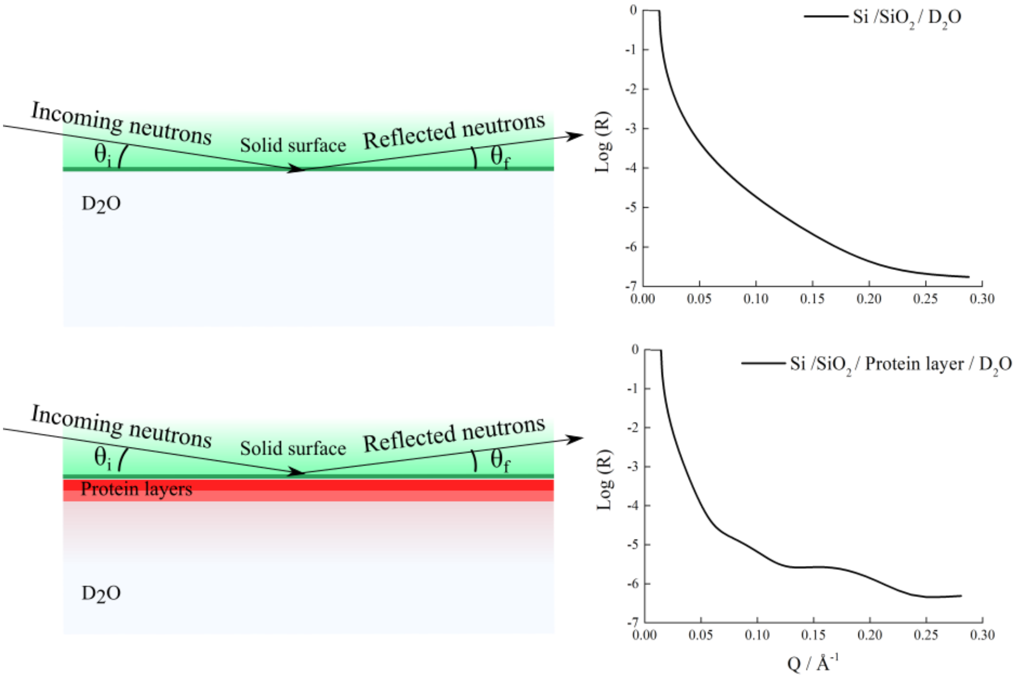


Figure S2. Schematic representation of neutron scattering experiment (left) and the corresponding measured signal (right).

Modelling reflectivity data can provide scattering length density of the layer adsorb to the surface. Knowing the scattering length density of the proteins, one can calculate the volume fraction of the proteins the in adsorbed layer and transfer that directly to the surface excess or the amount of proteins adsorbed to the surface by:

$$\Gamma = (t\, V_f)\, \rho_p \quad (2)$$

where t is the thickness of the adsorbed layer, $V_f$ is the volume fraction of proteins in the layer and $\rho_p$ is the mass density of proteins.

Reflectivity data is commonly shown as a function of changes in the magnitude of momentum transfer of neutrons before and after scattering. The momentum transfer perpendicular to the interface is given by:

$Q = (4\pi/\lambda)\sin(\theta_i)$ (3)

where λ is the wavelength of the neutrons.

Table S1. Properties of the materials used in this study.

| **Name** | **Formula** | **Formula Mass / g mol$^{-1}$** | **Density / g cm$^{-3}$** | **Molecular volume / Å$^3$** | **Scattering length Σ b / fm** | **ρ / $10^{-6}$ Å$^{-2}$** |
|---|---|---|---|---|---|---|
| Water | $H_2O$ | 18 | 0.9975 | 30 | -1.7 | -0.56 |
| Heavy water | $D_2O$ | 20 | 1.105 | 30 | 19.1 | 6.35 |
| Silica | $SiO_2$ | 60.08 | 2.16 | 46.2 | 15.8 | 3.41 |
| Silicon crystal | Si | 27.9 | 2.32 | 20.04 | 4.1 | 2.07 |
| Alumina | $Al_2O_3$ | 101.96 | 3.98 | 42.5 | 24.3 | 5.71 |
| *Moringa* protein in $H_2O$* | | ~ 9900 | 1.35 | 12400 | 1770 | 1.46 |
| *Moringa* protein in $D_2O$* | | ~ 10000 | 1.36 | 12400 | 3150 | 2.60 |

* Note that the values are measured and calculated for *Moringa oleifera* from Botswana and since the compositions are very similar, the same values have been used for all species of *Moringa* proteins.

**Neutron measurements**

The neutron reflectometry measurements for this study were made with the D17 reflectometer [2] in time-of-flight mode, with a wavelength range between 2 and 24 Å so that the data were recorded over a wide range of Q simultaneously. The average Q resolution was approximately 5% for the data presented in this study. Data were recorded at two incident angles of 0.7° and 3.2°, which allowed collection of reflectivity from about Q = 0.0075 to 0.25 Å$^{-1}$, although samples did not show a significant measurable signal beyond 0.2 Å$^{-1}$. The data was reduced to normalized reflectivity by using the data reduction program COSMOS [3].

**Analysis programs**

Reflectivity programs available on http://www.reflectometry.net/refprog.htm [4] were used for modelling reflectivity data from the substrate and the adsorbed protein layer. For bare surface characterization, the *wetdoc* program was used which allows simultaneous fitting of multiple contrast. Model fits showed that the surfaces were very similar in terms of roughness and thickness of oxide layer which was the case for the silicon substrates. Silicon surfaces were found to have formed a 10 to 12 Å thick layer of porous oxide (20-25 % water in the layer) with roughness < 6 Å on top. The sapphire surfaces were modelled with roughness up to 5 Å.

Reflectivity data from the proteins adsorbed to the surface was modeled using the *lprof* program (or *cprof* for multiple contrasts). The program allows modelling reflectivity from up to 4 layers with a fix scattering length density and further layer with a Gaussian, exponential, linear, or parabolic profile.

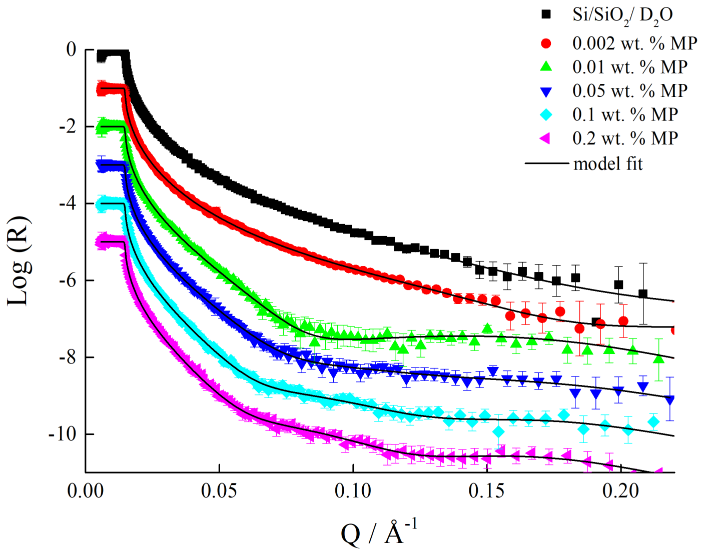


Figure S3. Reflectivity data and model fits from *Moringa peregrina* protein layer on silica ($SiO_2$) surface at different concentrations of proteins. Note that the curves are shifted from each other with one logarithmic unit to clarify the differences.

**Adsorption isotherm - model parameters**

For a comparison between the materials , we have fitted the adsorbed amount of each proteins at each surface to a Langmuir adsorption isotherm. The fitted parameters are shown in Table S2. $\Gamma_m$ represents the maximum amount adsorbed and K is the Langmuir adsorption constant, describing the kinetics of adsorption. Note that in the case of adsorption of *Moringa oleifera* and the silica surface, the number of data points were not sufficient to make this analysis.

Table S2. Langmuir Adsorption isotherm fitting parameters to the adsorbed amount of proteins onto silica and alumina surfaces.

| | **Alumina** | | **Silica** | |
|---|---|---|---|---|
| | $\Gamma_m$ (mg/m$^2$) | K | $\Gamma_m$ (mg/m$^2$) | K |
| ***Moringa oleifera* from Iran** | 1.8 | 257 | Not fitted | Not fitted |
| ***Moringa oleifera* from Botswana** | 5.1 | 74 | 4.6 | 71 |
| ***Moringa peregrina* from Iran** | 3.8 | 24 | 2.3 | 150 |

## Zeta potential measurements

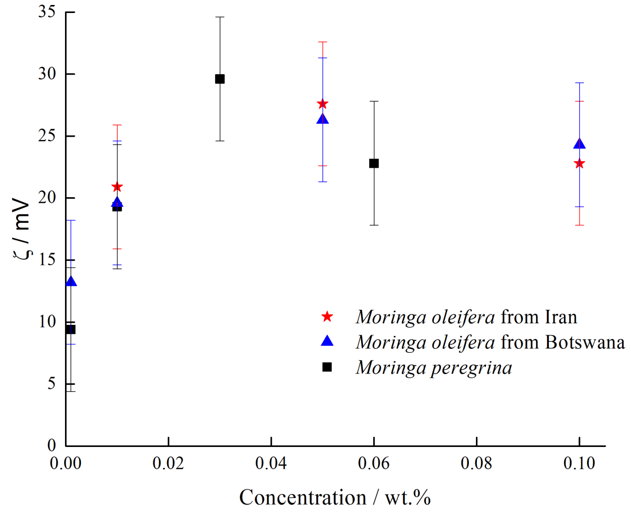


Figure S4. Zeta potential measured for different species in different concentrations.

## Effect of rinsing with water on the adsorbed *Moringa* protein layer

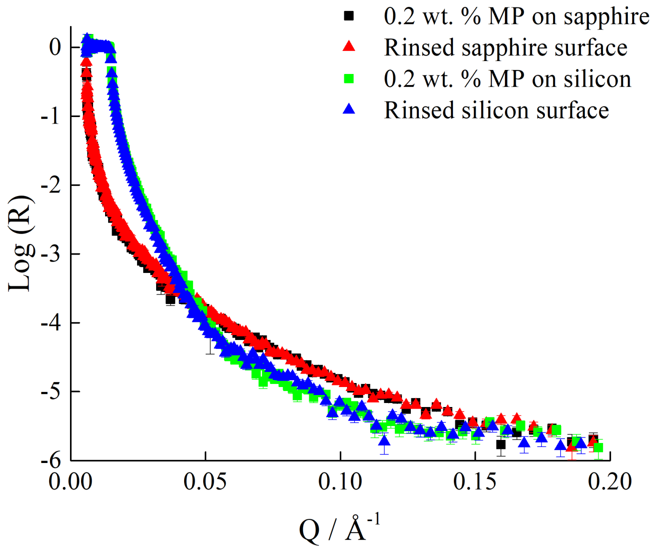


Figure S5. Effect of rinsing with water on the adsorbed of *Moringa peregrina* to silica and alumina surfaces.

## References


1. Thomas, R. K., Penfold, J., (1996). Neutron and X ray reflectometry of the interfacial system in colloid and polymer chemistry, *Current Opinion in Colloid & Interface Science*, 1, 1, 23-33.

2. Saerbeck T., Cubitt R., Wildes A., Manzin G., Andersen K. H., Gutfreund P. (2018). Recent upgrades of the neutron reflectometer D17 at ILL., J. Appl. Cryst. 51, 249-256.

3. Gutfreund P., Saerbeck T., Gonzalez M.A., Pellegrini E., Laver, M., Dewhurst C., Cubitt R. (2018). Towards generalized data reduction on a chopper-based time-of-flight neutron reflectometer., J. Appl. Cryst. 51, 606-615.

4. Rennie, A. R., (2018), Neutron reflectometry programs http://www.reflectometry.net/refprog.htm
